# Optimizing antimicrobial stewardship in the management of vertebral osteomyelitis: a decadal analysis at a central Taiwan Tertiary Teaching Hospital

**DOI:** 10.1017/ash.2025.160

**Published:** 2025-09-03

**Authors:** Changhua Chen

## Abstract

**Objectives:** Vertebral Osteomyelitis (VO) poses a formidable challenge, manifesting as infectious pathology affecting the vertebral body and intervertebral disc, marked by persistent back pain, unresponsiveness to conventional treatment, and elevated inflammatory markers. Timely therapy is crucial, emphasizing its impact on patient outcomes and aligning with antimicrobial stewardship principles. This study investigates the consequences of delayed appropriate antibacterial therapy on clinical outcomes, emphasizing its relevance within the context of optimizing antibiotic utilization. **Methods:** In a single-center cross-sectional study from January 2012 to December 2022, we focused on adult VO patients, diverging from standardized therapies to explore real-world treatment approaches. Criteria included clinical presentation, bacteriologic evidence, or imaging studies. Data, collected through the hospital’s clinical management software (HIS system), included demographic features, diseases, clinical history, laboratory findings, microbiological diagnoses, radiological details, complications, and outcomes. This analysis aims to provide nuanced insights into diverse management strategies and associated clinical outcomes related to VO while highlighting the pivotal role of antimicrobial stewardship. **Results:** The study involved 230 adult VO patients (mean age 64.7 years, male predominance 30.8%)(Table1). Positive microbiology cultures were found in 58.7% of cases, predominantly Gram-positive organisms. Patients with appropriate initial therapy had significantly lower severe back pain rates than those with inappropriate therapy (OR = 0.308, 95% CI 0.098, 0.971, p=0.044)(Table 2). **Conclusions:** Inappropriate antibiotics for VO treatment compromise symptomatic relief, emphasizing the pivotal role of antimicrobial stewardship. The study reveals significantly lower overall mortality rates and improved clinical symptoms with appropriate antibiotic therapy. In the context of VO’s severe illness and rapid deterioration, urgent enhancements in diagnostics and early appropriate antimicrobial therapy are crucial. This research provides substantive insights for optimizing VO management within the framework of antimicrobial stewardship, aiming to enhance patient outcomes and reduce healthcare system burdens.

